# Development of a mental health-related structural stigma measurement framework in the healthcare system setting: A modified Delphi study

**DOI:** 10.1371/journal.pone.0316999

**Published:** 2025-01-31

**Authors:** Dristy Gurung, Bhawana Subedi, Brandon A. Kohrt, Syed Shabab Wahid, Sauharda Rai, Graham Thornicroft, Petra C. Gronholm

**Affiliations:** 1 Transcultural Psychosocial Organization Nepal (TPO Nepal), Kathmandu, Nepal; 2 Centre for Global Mental Health and Centre for Implementation Science, Health Services and Population Research Department, Institute of Psychiatry, Psychology and Neuroscience (IoPPN), King’s College London, De Crespigny Park, London, United Kingdom; 3 Center for Global Mental Health Equity, The George Washington University School of Medicine and Health Sciences, Washington, DC, United States of America; 4 Department of Global Health, Georgetown University, Washington, DC, United States of America; 5 Center for Global Mental Health, Department of Population Health, London School of Hygiene & Tropical Medicine, London, United Kingdom; Erasmus University Rotterdam, NETHERLANDS, KINGDOM OF THE

## Abstract

**Introduction:**

There is a worldwide dearth in literature on the nature, causes, and consequences of structural stigma in mental healthcare. This study aimed to address this gap by exploring key components for measuring structural stigma in healthcare system settings.

**Methods:**

We used a modified Delphi method consisting of 3 rounds with global experts (stigma researchers, persons with lived experiences of mental health conditions (PWLEs), and policymakers). In the first round, indicators identified through a literature review (n = 39 studies) were appraised through expert consultation workshops with 22 panellists, including 54.5% women, 41% PWLEs, and 68.2% from low-and-middle income countries (LMICs). Round 2 (n = 53 panellists; 51% women, 8.3% PWLEs, and 56.6% from LMICs) involved ranking indicators through an online survey, and Round 3 (n = 58 panellists; 46% women, 21.7% PWLEs, and 60.4% from LMICs) involved re-ranking the results from Round 2. Smith’s salience index was calculated to measure consensus and Kendall’s coefficient of concordance to determine the degree of agreement. Narrative opinions and feedback from panellists during all three Delphi rounds were also sought.

**Results:**

A list of indicators within five core measurement domains was identified in Round 1. Round 2 results were heterogeneous as indicated by the low to moderate salience of most indicators. Round 3 resulted in 4–5 indicators in each domain, that were ranked as highly salient by the expert panellists. Experts also provided narrative feedback on the definition of structural stigma, barriers to its measurement, domain-specific comments, and indicators-specific comments.

**Conclusion:**

The framework aids in defining mental health-related structural stigma in healthcare and framing it in terms of inequities within healthcare system structures. These structures result in negative experiences of PWLEs and limit their access to quality healthcare. This conceptualization, informed by PWLE and stakeholders in LMICs, makes it easier to measure structural stigma and monitor changes in diverse healthcare settings around the world.

## Introduction

Stigma against people with lived experience of mental health conditions (PWLEs) can be conceptualized as an important but complex phenomenon that is fundamentally interlinked and functions at multiple levels (e.g. personal, public, and structural), which creates inequities in the healthcare systems [1]. Unfortunately, most stigma research has ignored these interlinkages and multi-level mechanisms of stigma and has focused mostly on internalized and interpersonal interactions [2]. There is a dearth in the literature on the nature, causes, and consequences of *structural stigma*, such that prominent stigma researchers have termed it “a dramatic shortcoming” in stigma research [3]. Various attempts have been made to explain the concept and processes of structural stigma. Corrigan and colleagues [4] divided structural discrimination into ‘intentional’ institutional discrimination that manifests as rules, policies, and procedures of organizations of power where they purposefully restrict the rights and opportunities; and ‘unintentional’ discrimination where the policies have consequences that restrict the opportunities in unintended ways. Similarly, other studies have tried to shed light on structural forms of stigma by documenting the discrimination in legal policies and provisions [5–7].

Conceptualizations of structural stigma come from frameworks outside healthcare systems, such as institutional/systemic racism [8] and discrimination against sexual and gender minorities [9]. Drawing from these theories and insights, Hatzenbuehler and Link (2014) proposed the following definition of structural stigma: “societal-level conditions, cultural norms, and institutional policies that constrain the opportunities, resources, and wellbeing of the stigmatized” [10]. This definition has facilitated Hatzenbuehler and colleagues to operationalize and measure structural stigma in research studies as in laws and policies and aggregated measures of social attitudes [10]. However, challenges have been highlighted in the measurement of structural stigma and discrimination, such as- scarcity of data sources and measurement tools for structural stigma, lack of variability within structural stigma making it difficult to study structural stigma within smaller geographic locations, lack of statistical power to detect changes in its effects [11], and its lack of application in low- and middle-income countries (LMICs) around the world [12]. Similarly, aggregated public attitudes and discrimination towards PWLEs may not be reflective of public attitudes relevant to structural discrimination [2]. An aggregate public attitude towards PWLEs may be positive, but attitudes towards financial resource allocation by governments to mental health may be different and less favourable. These conceptualizations of structural stigma also fail to capture the processes and mechanisms of structural stigma within local systems or settings, such as healthcare facilities within the health systems [13].

Livingston et al. [14] have focused on measuring structural stigma in healthcare contexts for PWLEs and have conceptualized structural stigma as a lack of access to care and poor quality of care. Based on this conceptualization, they proposed a framework consisting of six assessment domains (resource distribution, denial of care, fragmented care, practitioner practices, negative experiences, and coercive approaches), six methodological considerations (participatory, intersectional, multi-method, cross-levels, longitudinal, and outcomes), and six data sources (PWLEs, healthcare providers, health institutions, health insurers, governments, and legal systems). This framework provides options and approaches for documenting structural stigma so that changes over time and between healthcare contexts can be assessed. The framework also helps in understanding structural stigma as not just a conceptualization of the macro-level functions (legal, political, and economic structures), but also involving meso-level structures (institutions and professionals that form the structures). Such conceptualization is also seen in other studies of structural stigma in healthcare [15]. Although the framework by Livingston seems comprehensive and is developed through an in-depth review of the literature, its utility and comprehension by stakeholders working on reducing health-systems stigma are yet to be examined globally.

This study aims to explore the understanding of structural stigma in the context of healthcare systems among stakeholders (stigma researchers, PWLEs, policy makers, and stigma program implementer from high-income countries and LMICs) and identify the most important factors when measuring mental health-related structural stigma in this setting. These factors are used to distinguish indicators for the measurement of structural stigma in healthcare systems, building on the framework proposed by Livingston.

## Methods

This study used a modified Delphi method involving three rounds of data gathering and feedback from an international group of experts. Specifically, we sought to identify key indicators to measure mental health-related structural stigma in healthcare system settings, sort them into the measurement domains adapted from the framework proposed by Livingston, and rank the indicators in each domain. Ethical approval for this study was granted by the Psychiatry, Nursing, and Midwifery Research Ethics subcommittee at King’s College London (Project ref: HR/DP-21/22-24921) and Nepal Health Research Council (registration no: 132/2023). For the round 1 consultation workshops, participants were asked to provide a signed consent form before the workshops after reading the information sheet and agreeing to participate. For the round 2 and 3 surveys, the information sheet and consent were included in the beginning of the survey, and only those who agreed to participate by clicking the relevant box could continue with the survey. No minors were included in the study.

### Delphi method

The Delphi method is widely used in various sectors including health to promote agreement and build consensus among expert groups (panellists) on issues of interest [16–18]. It is an iterative and structured method where the expert panellists are asked to respond to the issue of interest anonymously. This modified Delphi method consisted of 3 rounds. The modification in the Delphi method was done during the first round, where an initial list of indicators was developed through a literature review and expert consultation workshops. The modified Delphi method was chosen because it allowed panellists to discuss what mental health-related structural stigma and discrimination mean in the healthcare system. This is due to a lack of common understanding and literature around it and the relative newness of the field [15].

#### Round 1: Development of measurement framework

The first round of the modified Delphi study consisted of developing a framework for the measurement of structural stigma and discrimination in healthcare systems through a literature review and expert consultation workshop.

*Identification of literature and experts*. Literature related to structural stigma and discrimination in mental healthcare was identified through a *non-systematic* literature review. A search of the literature was carried out using the terms “structural” AND “stigma” OR “discrimination” AND “mental illness” OR “mental health” in multiple databases: Web of Science, PsychINFO, and Google Scholar. The search was limited to articles published in English and the top 40 hits in each database were reviewed by DG and BS and their bibliographies were checked for cross-references.

PWLEs and stigma researchers/academics were recruited for the consultation workshop as expert panellists. The focus on researchers/academics and PWLEs for the consultation was mainly due to the aim of the workshops to explore conceptual understanding of structural stigma and identify indicators that mattered most. The panellists for the consultation workshop were identified through the INDIGO Partnership Research Programme [19] and Reducing Stigma among Healthcare Providers (RESHAPE) study [20]. The two projects were specifically chosen because both the projects focused on mental health-related stigma reduction and had the involvement of stigma researchers/academics and PWLEs.

*Process of Round 1*. Identified literature from databases and cross-references were reviewed by authors (DG & BS) to list indicators that were suggested and/or used to assess mental health-related structural stigma and discrimination. The list of indicators was then categorized into groups based on common themes and sorted into the domains in the framework proposed by Livingston on the measurement of structural stigma and discrimination in mental healthcare systems [14].

Four consultation workshops were carried out between April and June 2023. Three were online consultation workshops with 22 international experts including stigma researchers, program implementers, policy makers, and PWLE advocates (3 to 7 participants per workshop, discussions lasting between 1–1.5 hours). Workshops were recorded and conducted in English. Workshops consisted of free listing exercises of indicators for measurement of structural stigma and discrimination in the mental healthcare system using an online tool for free-listing and rating (https://easyretro.io/). Experts were then asked to rank the list of indicators identified through the free listing and sort them into the domains proposed by Livingston [14]. Understanding of structural stigma was explored during Round 1 of the Delphi exercise. Participants were asked to critique and disaggregate the definition of structural stigma provided by Hatzenbuehler and colleagues [10]–“societal level conditions, cultural norms, and institutional policies that constrain the opportunities, resources, and wellbeing of the stigmatized” and provide examples of mental health related structural stigma they have experienced or seen within the healthcare system settings.

A separate face-to-face workshop with 8 PWLEs from Nepal was conducted on June 25^th^, 2023 after approval from local ethical body. The discussions focused on their understanding of structural stigma and discrimination, experiences of structural stigma in healthcare systems, and how they think this form of stigma and discrimination can be measured. The discussions carried out in Nepali were audio-recorded and transcribed/translated by an experienced Nepali researcher in English.

Adaptations were made to the measurement Livingston framework based on the insights from Round 1, after which, the revised framework was shared with expert panellists in Round 2.

#### Round 2: Selection and ranking of indicators

*Expert selection and recruitment*. The criteria to participate in the Delphi study as expert panels were:

1. Leading health-related stigma researcher; AND/OR
2. Anti-stigma program implementer; AND/OR
3. Policymaker involved in the drafting or implementing of healthcare systems-related policies; AND/OR
4. A person with lived experiences of mental health conditions

The expert panel members were first identified from the mailing list of stigma researchers, anti-stigma program implementers, and policymakers who were part of the INDIGO network [19, 21] and the Lancet Commission on Ending Stigma and Discrimination in Mental Health [22]. They were approached via email to participate in the Delphi Survey after getting permission from the Principal Investigator and commission chairs. Additionally, they were asked to nominate individuals within their network who met the above criteria for inclusion. Thus 110 experts were identified and approached through this method. This also included panellists involved in Round 1 of the study.

PWLEs of mental health-related stigma were sent a link for the Delphi survey to which they could respond anonymously. The emails were sent by the president of the Global Mental Health Peer Network (GMHPN) to their network members. The GMHPN is an international organization led by people with lived experiences and consists of more than 300 network members from 45 countries around the globe [23]. Round 2 survey was active from 20^th^ June to 12^th^ July 2023 and completed by 53 panellists.

*Development of the survey and administration process*. The Qualtrics XM platform [24] was used for the development of the online survey. The survey consisted of study consent, demographic questions, and a list and description of indicators for the domains identified from Round 1 within each of the 5 domains. Panellists could also add up to 3 further indicators to the list if they felt these aspects were missing. Panellists were asked to select up to 5 indicators (from the list of indicators, including any new ones they added) in each domain. These were then ranked according to three criteria: 1) Appropriateness (whether the indicators were meaningful or appropriate in capturing structural stigma in a mental healthcare setting); 2) Usefulness (whether the indicators were sensitive to change and useful in comparing from one setting to another); and 3) Feasibility (whether the information for the indicators was feasible to collect). At the end of each domain, any additional comments could be added via an open-ended field. The participants were given options to complete the survey anonymously or provide email address for further communications regarding the study if required.

#### Round 3: Re-ranking of indicators

For the third round, we compiled the summary results of Round 2 that included the mean rank and average Smith’s Salience (of 3 criteria- appropriateness, usefulness, and feasibility) of the top 5 indicators for each domain. Everyone approached for Round 2 was sent the survey link to Round 3 irrespective of their participation in Round 2. Panellists were asked if they agreed with the summary results, and if not, they were asked to re-rank the indicators from the full list of indicators (including the indicators added by participants in Round 2). Additionally, panellists were asked an open-ended question on any comments regarding each domain. A specific question was added on the appropriateness of measurement domain 3 (‘aggregate stigmatizing attitude and practices of individuals affiliated with the health systems’) to measure structural stigma, in response to queries raised in Round 2 feedback. Round 3 survey was active from 10^th^ September to 4^th^ October 2023. Similar to Round 2, the participants were provided with option to complete survey anonymously or opt to provide their email for future correspondence on the topic.

### Data analysis

#### Round 1: Development of measurement framework

The indicators identified through the literature review were listed in an Excel sheet and the frequency of citation of the indicators was listed. The qualitative transcripts from the consultation workshops with PWLEs and stigma experts were coded and charted in the Excel sheet using broad *a priori* themes: i) understanding of structural stigma and its definition; ii) barriers and facilitators to the measurement of structural stigma; iii) measurement domain specific comments and feedback; and iv) indicator-specific comments and feedback.

The free-listed indicators during the consultation workshops were sorted into the domains provided by Livingston [14] and the experts themselves. The domains were modified based on the feedback from experts during the workshops. Any additional indicators identified during discussions were supplemented by the indicators identified during the literature review. These indicators too were then sorted into the adapted domains after the consultation workshops.

#### Round 2: Selection and ranking of indicators

For the second-round survey, as panellists were asked to select up to 5 indicators from a list of indicators and rank them, panel consensus was not just limited to the ranking of indicators but also the item selection. Hence, Smith’s Salience Index (Smith’s S) was used as a measure of consensus for the survey. Smith’s S is a function that considers the frequency of item selection and its average rank and can be defined as: S = (∑ (L_i_ − R_ij_ + 1) / L_i_) / N where R_ij_ is the rank of the item “j” in the list given by respondent i, L_i_ is the length of respondent i’s list, and N is the total number of lists in the sample [25]. Smith’s S provides a value between 0 and 1 and has been used as a measure of consensus in previous Delphi studies where values closer to 1 were considered higher consensus [26]. Smith’s S above 0.5 were “highly salient”, 0.3–0.49 was “moderate salience”, and below 0.3 was “low salience” [26].

FLARES software for cultural analysis [27] was used to analyse the data. The dataset from Qualtrics software was downloaded and entered in the FLARES software, where mean rank, frequency of selection, and Smith’s S were analysed statistically for each of the 3 criteria (appropriateness, usefulness, feasibility) in each domain. A similar analysis was conducted on the dataset with responses only from panellists identifying as PWLEs. The Smith’s S across the 3 criteria were aggregated using average mean giving equal weights to all 3 criteria. A list of the top 5 ranked indicators (as determined by the average salience) in each domain was created and shared with the panellists for re-ranking in Round 3. Open-ended narrative feedback on the survey was reviewed and synthesized, based on which further follow-up questions were added in Round 3.

#### Round 3: Re-ranking exercise

At the end of Round 3, rather than the frequency of item selection, panellists were asked to re-rank the list of full indicators (including the ones added by panellists in Round 2) if they disagreed with the ranking from Round 2. This was done because of moderate to low salience seen in Round 2 due to variability in item selection. Hence, the calculation of Smith’s S considered the ranking of the indicators as the frequency of selection for all indicators in this round was 1. Similarly, to assess the agreement between panellists in the ranked order of items, Kendall’s concordance of coefficient (Kendall’s W) was calculated. Kendall’s W is a non-parametric test for rank correlation that ranges from 0 (no agreement) to 1 (complete agreement) [28]. The Kendall’s W test was conducted in IBM SPSS for Windows version 29.0 [29]. Similar to Round 2, a sub-analysis of responses from PWLEs was conducted to highlight any differences in opinions. Open-ended feedback on the survey was reviewed thematically and reported in a narrative format.

## Results

### Panellist demographics

Table 1 shows the demographics of experts in each round of the Delphi study. Twenty-two experts participated in the expert consultation (54.5% women). Eight out of nine PWLEs in the consultation workshop were from rural Nepal who were working as advocates in a stigma reduction program called RESHAPE [20]. PWLEs were mostly representatives from LMICs in both Round 2 (60%) and 3 (66%). In Round 2 of the Delphi exercise, 53 participants completed the survey 51% women). Fifty-eight panellists completed the Round 3 survey (46% women).

**Table 1:** Demographics of experts in each Delphi round.

Demographics	Round 1: Consultation workshop n (%)	Round 2: Survey n (%)	Round 3: Re-ranking survey n (%)
**Gender**	N = 22	N = 53	N = 58
Female	12 (54.5%)	27 (51.0%)	28 (46.0%)
Male	10 (45.5%)	25 (47.0%)	26 (45.0%)
Non-binary	-	1 (1.0%)	2 (3.0%)
Prefer not to answer	-	-	2 (3.0%)
**Type of expertise** *			
Expert by experience/PWLE advocate	9 (41.0%)	5 (8.3%)	15 (21.7%)
Stigma researcher/academic	12 (54.5%)	42 (70.0%)	43 (62.3%)
Policymaker/implementer	1 (4.5%)	13 (21.7%)	11 (16.0%)
**Region**			
South Asia	10 (45.4%)	16 (30.2%)	19 (33.0%)
Latin America and the Caribbean	-	1 (1.9%)	-
East Asia and the Pacific	-	6 (11.3%)	8 (14.0%)
Sub-Saharan Africa	3 (13.6%)	5 (9.4%)	7 (12.0%)
North Africa and the Middle East	2 (9.1%)	2 (3.7%)	2 (3.0%)
North America	2 (9.1%)	4 (7.5%)	5 (9.0%)
Europe and Central Asia	5 (22.8%)	17 (32.1%)	14 (24.0%)
Australia and New Zealand	-	2 (3.7%)	3 (5.0%)
**Country classification (World Bank)**			
High-Income	7 (31.8%)	23 (43.4%)	23 (39.6%)
Low and Middle income	15 (68.2%)	30 (56.6%)	35 (60.4%)

*Respondents could select multiple options

### Literature review and expert consultations (Round 1)

Through the literature review, we identified 39 papers that discussed structural discrimination in the healthcare system, which was not limited just to mental healthcare. Some studies focused on discrimination in healthcare for LGBTQ+ communities while some on racial minorities. A list of indicators used or provided as an example of structural forms of discrimination was listed and grouped (See *supporting document* S1 Table for the list of papers and indicators identified). Most of the papers identified discriminatory laws, legislations, and policies as key representations of structural stigma and discrimination (n = 28), followed by cultural norms, practices, and aggregated attitudes (n = 19), inequitable or lack of resource allocation and funding (n = 14), lack of access to care/services (n = 10), and lack of parity in benefits (coverage in health insurance) (n = 6).

Similarly, a list of indicators was generated during the free listing and sorting exercise in the expert consultation workshops. Panellists identified a need for an additional domain on discriminatory laws and legislations that were not captured by the Livingston framework. Similarly, experts suggested that coercive care, fragmented care, and denial of care could all mean poor quality of care and recommended merging the domains to simplify the framework.

These exercises resulted in a framework consisting of 5 domains, with each comprising a unique list of indicators for each domain. See Table 2 for the full list of indicators in each domain and its source of identification in Round 1.

**Table 2:** Indicators identified through literature review and expert consultations (Round 1).

Domains and indicators	Literature Review (n = 39)	Consultation workshop with stigma researchers, academics/policy makers (n = 13)	Consultation workshop with PWLEs (n = 9)
**Domain 1: Discriminatory legal framework and policy environment**			
1. Availability of mental health policy and action plans	✓	✓	-
2. Exclusion of mental health from Universal Health Coverage	-	✓	-
3. Exclusion of mental health from other national health policies and programs	✓	✓	-
4. Lack of space for People with Lived Experiences (PWLE) involvement in policy/program development	✓	✓	✓
5. Discriminatory language or provision in mental health policy	✓	✓	✓
6. Lack of coverage in national /community health insurance policies	✓	✓	✓
7. Exclusion of mental health from disability or other social-welfare policies	✓	✓	-
8. Differential rules or guidelines for PWLEs to access health services	✓	✓	✓
9. Differential rules or guidelines for storage/access of information of PWLEs	-	✓	-
10. Underfunded mental health policies	✓	✓	✓
**Domain 2: Stigmatizing system infrastructure and resource allocation**			
1. Insufficient funding for mental health services & programs	✓	✓	✓
2. Unavailability of trained mental health human resources	✓	✓	✓
3. MH indicators not included in national health information system	✓	✓	-
4. Differential quality of space/infrastructure for mental health compared to other health services	✓	✓	✓
5. Unaffordable services compared to other chronic conditions	-	✓	-
6. Insufficient funding for mental health research	-	✓	-
7. Spaces that are undignified or non-conducive to recovery	✓	✓	✓
8. Unavailability of mental health medications at the health facilities	✓	✓	✓
9. Stigmatizing messages against mental health in information/education materials that are available	-	✓	-
10. Number of mental health training conducted per year for general health workers	-	✓	-
11. Mental health recording/reporting is unsystematic and ad hoc compared to other health services	-	✓	-
12. Systematically fewer hours allocated for mental health training compared to other health training	✓	✓	-
13. Unsystematic/poor supply chain management of mental health medications compared to other medicines	-	✓	-
14. Unavailability of Information Education materials and guides for mental health	-	✓	-
**Domain 3: Aggregate stigma attitudes and practices of individuals within healthcare systems**			
1. Negative aggregate attitude/behaviour of health and other staff towards PWLEs	✓	✓	✓
2. Health Workers not aware or knowledgeable about the human rights of PWLEs	✓	✓	✓
3. Less aggregate competency in dealing with MH patients	✓	✓	✓
4. Culture of not involving PWLEs in decision-making	-	✓	-
5. Culture of stigmatizing mental health staff by other professionals in the healthcare system	-	✓	-
6. Withholding information from PWLEs	-	-	✓
**Domain 4: Inequitable and poor quality of care**			
1. Involuntary/compulsory treatment of PWLEs	✓	✓	✓
2. Unavailability of evidence-based MH services	✓	✓	-
3. Separation of MH services from Primary health or basic health services	✓	✓	✓
4. Lack of multi-sectoral collaboration within health systems for mental health compared to other health conditions	-	✓	-
5. Lack of a clear referral pathway system	✓	✓	-
6. Lack of sufficient outpatient care for people with severe MH conditions	✓	✓	✓
7. PWLEs are not able to easily access disability services and grants	-	-	✓
8. Paternalistic/non-collaborative approaches	✓	✓	✓
9. Lack of access to rehabilitation services for PWLEs	-	-	✓
10. Segregated health and social care systems compared to other health conditions	✓	✓	✓
11. Interaction with justice/security during treatment	-	✓	-
12. Exclusion of PWLEs from screening services	-	✓	-
13. Delay in onset of mental health condition to start of treatment	✓	✓	-
14. Diagnostic & Treatment overshadowing	✓	✓	✓
15. Medication errors for mental health care compared to general health care	-	✓	✓
**Domain 5: Negative experiences of PWLEs**			
1. PWLE low satisfaction of care received for mental health	✓	-	✓
2. PWLE negative interaction with Health workers/administrators	✓	✓	✓
3. PWLE has higher Out-of-pocket expenses for MH services compared to other health services	✓	✓	✓
4. PWLE lack of ease of access of mental health services vs physical services	✓	✓	✓
5. PWLE insufficiently informed about their condition or treatment	✓	✓	✓
6. PWLE experience of undignified treatment process	✓	✓	✓
7. PWLE lack of ease of access to social services when needed	✓	-	✓
8. PWLE feeling devalued and infantilized by Health Workers/admins	✓	✓	✓
9. PWLE experience of hasty referrals or no referrals (even when needed)	-	-	✓
10. PWLE experience of being hastily diagnosed and treated	✓	-	✓

### Ranking exercise (Round 2)

Table 3 provides the top 5 average salient indicators for each domain within the mental healthcare system-related structural stigma measurement framework with its consecutive salience, average rank, and frequency of selection within each criterion (appropriateness, usefulness, feasibility). The full list of indicators and their ranking, Smith’s S, and frequency of selection for all 3 criteria can be seen in *Supporting document* S2 Table.

**Table 3:** Ranking of indicators based on appropriateness, usefulness, and feasibility (Round 2).

Top 5 indicators in each domain	Appropriate			Useful			Feasible			Average Smith’s S*
	Smith’s S	Frequency (%)	Average Rank	Smith’s S	Frequency (%)	Average Rank	Smith’s S	Frequency (%)	Average Rank	
**Domain 1: Discriminatory legal framework and policy environment**										
Unavailability of mental health policy and action plans**	0.49	61.5	1.75	0.49	63.5	1.93	0.63	76.9	1.72	**0.54**
Exclusion of mental health from Universal Health Coverage	0.36	53.8	2.43	0.25	38.5	2.35	0.38	55.8	2.27	**0.33**
Exclusion of mental health from other national health policies and programs	0.31	48.1	2.44	0.35	57.7	2.63	0.29	50	2.65	**0.32**
Lack of involvement of People with Lived Experiences (PWLE) in policy/program development**	0.37	55.8	2.43	0.29	50	2.65	0.25	44.2	2.82	**0.31**
Discriminatory language or provision in mental health policy**	0.21	32.7	2.41	0.29	46.2	2.37	0.26	44.2	2.39	0.26
**Domain 2: Stigmatizing system infrastructure and resource allocation**										
Insufficient funding for mental health services & programs**	0.72	82	1.48	0.59	76	1.92	0.46	62	2.03	**0.59**
Unavailability of trained mental health human resources**	0.35	54	2.33	0.36	52	2.19	0.32	50	2.44	**0.35**
MH indicators not included in national health information system**	0.21	38	2.94	0.21	40	2.9	0.32	46	2.17	0.24
Unaffordable services compared to other chronic conditions**	0.20	32	2.62	0.18	28	2.57	0.21	28	2.21	0.19
Differential quality of space/infrastructure for mh compared to other health services	0.27	40	2.4	0.13	20	2.6	0.19	30	2.53	0.19
**Domain 3: Aggregate stigma attitude and practices of individuals within healthcare systems**										
Negative aggregate attitude/behavior of health and other staffs towards PWLEs**	0.58	76	1.94	0.59	78	1.81	0.49	72	2.13	**0.55**
HWs not aware or knowledgeable about human rights of PWLEs**	0.42	74	2.65	0.45	72	2.33	0.53	82	2.19	**0.47**
Less competency in dealing with mh patients	0.36	62	2.61	0.47	70	2.17	0.42	60	2	**0.42**
Culture of not involving PWLEs in decision-making **	0.49	68	2.03	0.34	66	2.76	0.33	52	2.27	**0.39**
Culture of stigmatizing mental health staff by other professionals in healthcare system **	0.29	58	2.96	0.27	44	2.54	0.24	40	2.3	0.27
**Domain 4: Inequitable and poor quality of care**										
Involuntary/compulsory treatment of PWLEs**	0.35	44	1.90	0.27	43	2.52	0.32	42	2	**0.31**
Unavailability of evidence-based MH services	0.27	42	2.52	0.29	41	2.2	0.31	49	2.58	0.29
Separation of mh services from Primary health or basic health services	0.26	37	2.22	0.26	43	2.52	0.34	49	2.20	0.28
Lack of multi-sectoral collaboration within health systems for mental health compared to other health conditions	0.34	53	2.5	0.32	51	2.52	0.20	32	2.56	0.28
Lack of clear referral pathway system	0.19	37	3.05	0.18	37	3.16	0.24	42	2.62	0.21
**Domain 5: Negative experiences of PWLEs**										
PWLE low satisfaction of care received for mh**	0.33	45	2.18	0.35	47	1.82	0.51	65	1.81	**0.39**
PWLE negative interaction with HW/administrators**	0.49	65	2.03	0.31	49	2.45	0.29	49	2.58	**0.36**
PWLE higher Out of pocket expenses for mh services compared to other health services**	0.24	45	3.09	0.37	53	2.11	0.34	44	1.77	**0.32**
PWLE lack of ease of access of mh services vs physical services	0.28	45	2.41	0.33	49	2.08	0.27	46	2.47	0.24
PWLE insufficiently informed about their condition or treatment	0.31	49	2.54	0.19	35	2.76	0.18	26	2.23	0.23

Bold average Smith’s S indicates moderate to high salience.

* Mean average Smith’s Salience Index of all 3 criteria (appropriate, useful, feasible)

** Indicators that had moderate to high salience (above 3) among PWLEs (n = 5)

With a few exceptions, most indicators had moderate to low salience indicating great heterogeneity in the endorsement of indicators. In the discriminatory legal and policy framework domain (domain 1), “unavailability of mental health policy and action plans” was ranked the highest across all 3 criteria (*S =* 0.54). Although the “lack of involvement of PWLEs in policy/program development” was the second most salient indicator in appropriateness (*S =* 0.37), it ranked low in usefulness (*S =* 0.29) and feasibility (*S =* 0.25). However, this indicator was endorsed as highly salient by PWLEs across all three criteria (*S =* 0.59).

In the “stigmatizing system infrastructure and resource allocation” domain (domain 2), “insufficient funding for mental health services and programs” was ranked highest in all 3 criteria by all panellists (*S =* 0.59). PWLEs specific ranking for the same indicators was also high (*S =* 0.68). “Unavailability of trained mental health human resources” was endorsed as moderately salient (*S =* 0.35) making it the only indicator to be endorsed as high or moderate salient in the domain by all panellists. Although “mental health indicators not included in national health information system” and “unaffordable services compared to other chronic conditions” were ranked low overall, they were endorsed as moderately salient by PWLEs (S = 0.35 for both indicators).

In domain 3 “aggregate stigma attitude and practices of individuals within healthcare systems”, “negative aggregate attitude/behaviour of health and other personnel in healthcare system towards PWLEs” was ranked the most salient by all panellists (*S =* 0.55) (PWLEs specific panellists ranking was also high (*S =* 0.66)). The indicator “culture of not involving PWLEs in decision-making process” was moderately salient overall (*S =* 0.39) but was endorsed as highly salient by PWLEs (*S =* 0.55).

For domain 4, as the number of indicators in the original list was higher (N = 15), saliency for the indicators was lower than other domains that had smaller list of indicators. The highest-ranked indicator in domain 4 was “involuntary/compulsory treatment of PWLEs” which was endorsed as moderately salient overall (*S =* 0.31) but as highly salient by PWLEs (*S =* 0.65). All other indicators had low salience overall, however, “lack of sufficient outpatient care for mental health” (*S =* 0.30) and “paternalistic/non-collaborative approaches (*S =* 0.30) were ranked as moderately salient by PWLEs.

For the final domain “negative experiences of PWLEs” (domain 5), “PWLE low satisfaction of care received for mental health” was ranked highest by all experts with moderate salience (*S =* 0.39) and high salience by PWLEs (*S =* 0.57). This was followed by “PWLE negative interaction with personnel within healthcare system” (*S =* 0.36), and “PWLE higher out-of-pocket expenses for mental health compared to other health services” (*S =* 0.32) with a moderate level of salience.

Although the survey allowed panellists to add new indicators in each domains, only two new indicators (“culture of ‘othering’ PWLEs” and “culture of non-disclosure of own mental illness among health workers”) were added in domain 3 which did not have high salience.

### Re-ranking exercise (Round 3)

Table 4 shows the top 5 indicators for each of the 5 domains with their average rank, salience, and degree of convergence of agreement as denoted by Kendall’s W and its P-value. See *Supporting document* S2 Table for full list of indictaors and their ranking for Round 3. Although there was moderate to lower consensus in Round 2 among the panellists as determined by the salience index, in round 3, most panellists agreed with the ranking of the indicators. The Smith’s Salience index in Round 3 improved as panellists were provided with the complete list of indicators and asked to re-rank them again if they disagreed with the Round 2 rankings, and so the frequency of selection was 100%. One third of panellists disagreed with the ranking of some of the indicators in each domain. Overall ranks of the indicators remained the same and there was overall strong agreement as shown by Kendall’s coefficient of concordance in all domains, which was above 0.8 (p<0.001).

**Table 4:** Re-ranking of indicators based on importance arranged by all experts and PWLEs only (Round 3).

	All panellists (n = 58)			PWLEs only panellists (n = 15)		
Top 5 indicators in each domain	Smith’s S	Average rank	Kendall’s W (p-value)	Smith’s S	Average rank	Kendall’s W (p-value)
**Domain 1 Discriminatory legal framework and policy environment**						
Unavailability of mental health policy and action plans	0.96	1.32	0.87 (<0.001)	0.93	1.6	0.82 (<0.001)
Exclusion of mental health from Universal Health Coverage	0.86	2.33		0.81	2.8	
Exclusion of mental health from other national health policies and programs	0.75	3.44		0.75	3.4	
Lack of involvement of People with Lived Experiences (PWLE) in policy/program development	0.71	3.91		0.74	3.6	
Discriminatory language or provision in mental health policy	0.61	4.84		0.64	4.5	
**Domain 2 Stigmatizing system infrastructure and resource allocation**						
Insufficient funding for mental health services & programs	0.97	1.32	0.88 (<0.001)	0.91	2.2	0.79 (<0.001)
Unavailability of trained mental health human resources	0.91	2.22		0.87	2.7	
MH indicators not included in national health information system	0.82	3.43		0.81	3.6	
Unaffordable services compare to other chronic conditions	0.75	4.5		0.78	4	
Differential quality of space/infrastructure for mental health compared to other health services	0.69	5.24		0.71	5.1	
**Domain 3 Aggregate stigma attitudes and practices of individuals within healthcare systems**						
Negative aggregate attitude/behaviour of health and other staff towards PWLEs	0.96	1.29	0.86 (<0.001)	0.97	1.2	0.84 (<0.001)
Health Workers not aware or knowledgeable about the human rights of PWLEs	0.86	2.20		0.83	2.46	
Less competency in dealing with mental health patients	0.77	3		0.78	2.93	
Culture of not involving PWLEs in decision-making	0.65	4.13		0.63	4.26	
A culture of stigmatizing mental health staff by other professionals in healthcare system	0.53	5.19		0.50	5.46	
**Domain 4 Inequitable and poor quality of care**						
Involuntary/compulsory treatment of PWLEs	0.96	1.56	0.83 (<0.001)	0.96	1.53	0.84 (<0.001)
Unavailability of evidence-based MH services	0.87	2.82		0.88	2.73	
Separation of mental health services from Primary health or basic health services	0.79	4.08		0.79	4.13	
Lack of clear referral pathway system	0.74	4.91		0.71	5.33	
Lack of multi-sectoral collaboration within health systems for mental health compared to other health conditions	0.72	5.13		0.74	4.86	
**Domain 5 Negative experiences of PWLEs**						
PWLE low satisfaction of care received for mental health	0.96	1.37	0.87 (<0.001)	0.96	1.33	0.84 (<0.001)
PWLE negative interaction with HW/administrators	0.87	2.25		0.88	2.2	
PWLE higher Out of pocket expenses for mental health services compared to other health services	0.76	3.31		0.76	3.4	
PWLE lack of ease of access of mental health services vs physical services	0.69	4.01		0.61	4.8	
PWLE insufficiently informed about their condition or treatment	0.58	5.12		0.59	5.06	

Corresponding to Round 2, we conducted a sub-analysis of the ranking with responses from PWLEs only. There were no differences in ranking in any of the domains among PWLEs as compared to the ranking by the full experts. The lowest agreement on ranking among PWLEs was seen in domain 2, however, the domain still had a significantly high level of agreement (Kendall’s W = 0.79).

### Narrative feedback

#### Understanding of structural stigma and its definition

The experts during the consultation workshops commented on the vagueness of societal-level conditions as not capturing anything substantial while some reflected that ‘cultural norms’ and ‘societal conditions’ could mean the same thing which is focused on stereotypes and their use in institutional practices. Everyone agreed that the ‘institutional policies’ in the definition were more specific and understandable, which may be why it is widely used as an indicator of structural stigma. However, they also cautioned that the definition does not include ‘lack of policy’, which in many cases reflects structural stigma.

Among the PWLEs in Nepal, it was difficult for them to comprehend the definition and concept of structural stigma, as they viewed stigma in terms of their personal experiences and negative interactions in the healthcare setting. However, almost all the PWLEs highlighted the lack of availability of medications and lack of trained mental health personnel in local healthcare settings as a key form of discrimination. Similarly, when talking about negative experiences, they shared the fear and confusion they faced due to the lack of information provided by staff at hospitals during their involuntary admission. *“I thought I was imprisoned in the hospital ward forever without any prospect of going back home…No one told me how long I was admitted for*. *I thought- I wasn’t like other ‘crazy’ people around me*, *I didn’t show such symptoms*. *So*, *I couldn’t understand why I was imprisoned with them*. *The fear and confusion made my mental health even worse” (Male*, *PWLE from Nepal*).

Other examples of structural stigma and discrimination shared during expert consultations were mainly policy-specific (e.g. lack of mental health policy or discriminatory policies), resource-related (e.g. lack of private space, unavailability of mental health-related medications, and lack of funding for mental health), and service-related (unavailability of mental health services at local health facilities and poor quality of mental healthcare compared to other health conditions). A psychiatrist shared how medical personnel such as nursing staff were reprimanded for bad behaviour by placing them in the mental hospital. Another expert highlighted structural discrimination in the health education of medical students. *“In medical examinations of emergency medicine*, *even if medical students fail mental health-related competency evaluations for mental health*, *you could still pass*. *However*, *that is not the case for other medical conditions”*. *(Female*, *Psychiatrist/stigma researcher)*. Similarly, district nurses refusing to visit homes of people with severe mental health conditions because they can walk and come to General Practice and only visit homes of people who are physically unable to walk was described as a discriminatory practice that could fall under structural stigma.

#### Barriers and facilitators to the measurement of structural stigma

One of the key barriers highlighted by the experts was the difficulty in measuring structural stigma due to the broadness of the concept and the difficulty in operationalization of the concept. Experts struggled to operationalize the ‘cultural norms’ aspect of the structural stigma. Experts mentioned that structural stigma takes a long time to translate into action and contrarily, any action targeting structural stigma takes a long time to show outcomes, which could be the reason for the dearth of research in the field. An expert suggested that the difficulty in operationalizing structural stigma could be because of the structural stigma itself. *“Structural stigma in itself means there is a lack of indicators and studies conducted on having the right measures*. *Or the workforce being limited in terms of time to do research or even think about structural forms of stigma and discrimination within healthcare systems or healthcare policies*.*” (Female*, *Stigma researcher)*

Another challenge highlighted in the measurement of structural stigma was the comparative aspect of mental health-related structural stigma. An expert commented- *“For me*, *structural stigma needs to have a sense of ’differential’ impact compared to something else*. *I chose ’unavailability’ of medicines/mental health indicators because we know that other health conditions have those and so unavailability indicates something that is differential” (Female*, *Stigma researcher)*. Some experts discussed possibilities of comparison with chronic or long-term health conditions while others cautioned against such medical comparisons and advocated for social disability framework.

#### Domain-specific feedback

One of the key points raised by experts in the consultation workshops (Round 1) was the inter-connectedness of the Livingston domains. The participants reflected that it was difficult to sort indicators into domains because they were interrelated as causes and consequences. Indicators within one domain could lead to another and might fit into both domains. Experts reflected that it would make better sense to merge some of the domains such as “denial of care” and “coercive approaches” to make it simpler and add other domains to accommodate indicators like lack of mental health policies or discriminatory policies.

During the online surveys (Rounds 2 and 3), most domain-specific comments were provided in domain 3 (aggregate stigma attitude and practices of individuals within healthcare systems). Panellists commented on the domain not being reflective of structural stigma and asked for the removal of the domain as it *“refers to attitudes and behaviours of individuals*, *and to call this structural stigma seems self-contradictory” (Male*, *Stigma researcher)*. In Round 3, participants were asked if they agreed to the removal of the domain from the framework and to provide reasons for their response. Sixty-four percent of the panellists (80% PWLEs) disagreed with the statement that the domain did not reflect structural stigma. Almost all the experts stated that the individuals make up the structure and so are the enablers of discriminatory culture and practices within the structure. A panellist remarked- *“Attitudes and behaviour of professionals reflect organizational culture so while they are measured at the interpersonal level can be seen as an aspect of structural stigma; the same people might behave quite differently in another organization and given better training” (Female*, *stigma researcher)*. Alternatively, some experts suggested that the domain could be mentioned as supplementary to the other domains as a cause or consequence of structural stigma rather than fitting it within the framework, while others (mostly PWLE panellists) advocated for fitting it within the framework as a pathway that reflects structural forms of discrimination.

#### Indicator-specific feedback

Feedback on making indicators specific, adding indicators that are from PWLE’s perspective, and feedback on the relevance or irrelevance of certain indicators in the domains were some examples of indicator-specific feedback provided by panellists.

Most panellists commented on the indicator “involuntary/compulsory treatment of PWLEs” in domain 4. Some panellists reflected that the indicator might not be reflective of structural forms of discrimination, as it may be necessary in some cases to treat people involuntarily. A PWLE panellist mentioned *“Involuntary admission should be nuanced*. *As a PWLE*, *I’m grateful to my parents for involuntarily getting me to treatment when I was in a manic episode*. *Involu ntary treatment is sometimes the solution than the problem” (Female*, *PWLE)*.

Similarly, feedback was received on the importance of indicators such as “unavailability of medications”, “underfunding of the mental health sector”, and “differential quality of space for mental health”, especially for LMICs. A panellist mentioned- *“It will be difficult to measure the quality of the space*, *but stock-outs or non-availability of essential medications for mental health is easy to measure*. *Also*, *medication access is highly valued by people with lived experience in studies from Africa” (Female*, *Stigma researcher)*.

## Discussion

**Fig 1:**
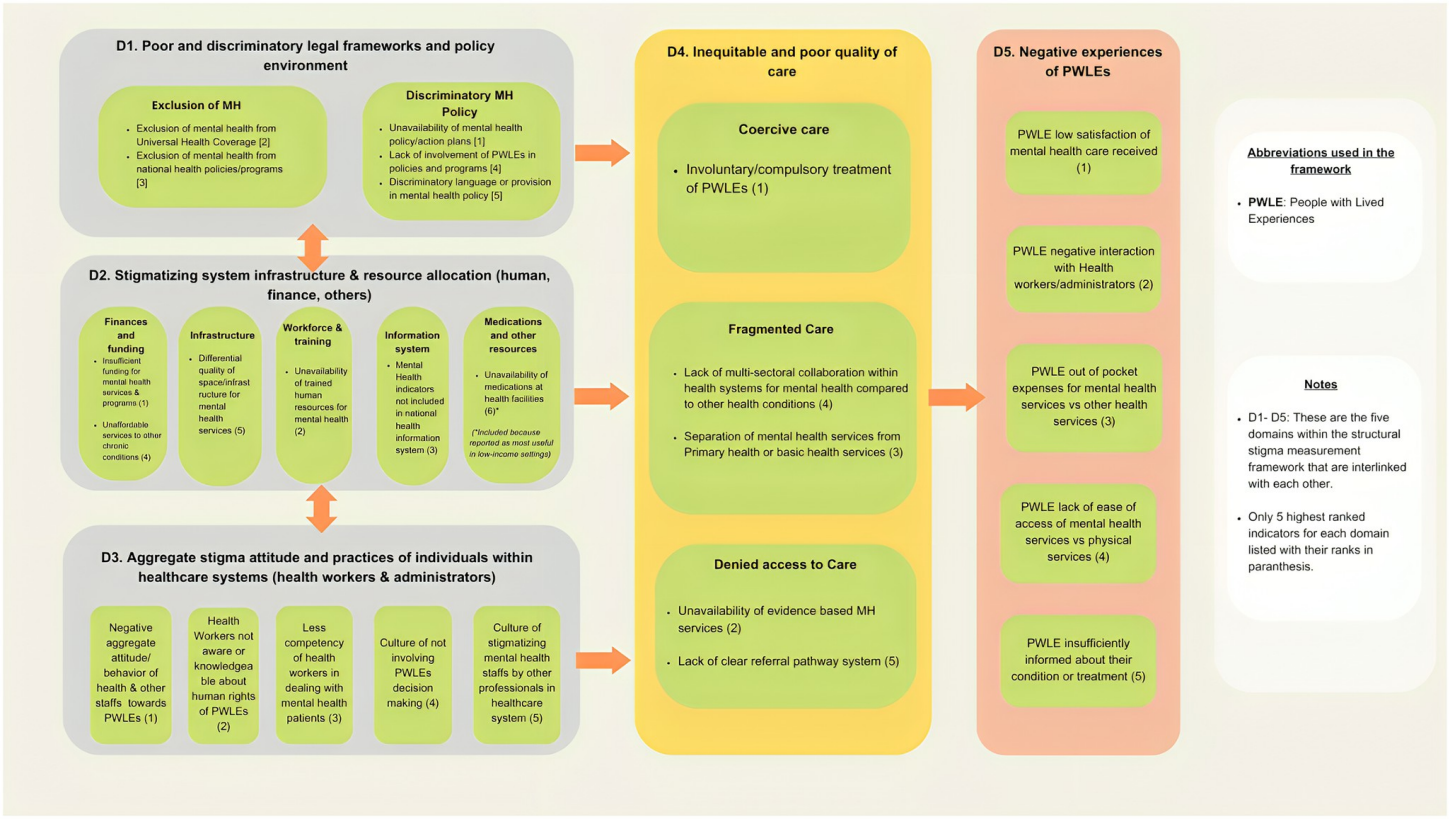
Structural stigma measurement framework in the healthcare system.

This study engaged researchers and PWLE around the world, including many stakeholders from LMICs to identify indicators that matter most to stakeholders in the measurement of mental health-related structural stigma in healthcare settings. A modified Delphi method consisting of 3 rounds of consultation workshops and feedback was conducted to identify and rank indicators. Through the study, a measurement framework consisting of 5 domains (1. discriminatory legal framework and policy environment; 2. stigmatizing system infrastructure and resources; 3. Aggregate stigma attitudes and practices of individuals within healthcare systems; 4. inequitable and poor quality of care; and 5. negative experiences of PWLEs) and a list of salient indicators were identified. Fig 1 shows the domains and list of indicators identified through the Delphi process. The framework is an expansion and adaptation of the measurement framework proposed by Livingston for the measurement of structural stigma in healthcare settings [14].

The proposed framework has several advantages in understanding and measuring mental health-related structural stigma in healthcare contexts. **First**, it provides conceptual clarity of structural stigma and helps to find consensus in the operationalization of the structural stigma definition when applied to healthcare system settings. One of the shortcomings of the structural stigma research is the lack of universal understanding of the concept resulting in heterogeneity in definitions and measures that has widened the conceptual gap further [15]. The scattered conceptual understanding can also be seen in our findings from the Round 2 survey where the selected indicators and their ranking varied across the panellists resulting in most indicators having low to moderate salience. The experts during the consultation workshops also found it difficult to dismantle the components within the most cited definition by Hatzenbuehler and Link that included ‘societal conditions’, ‘cultural norms’, and ‘institutional policies’ that restricted the rights and opportunities of the stigmatized individuals [10]. The institutional policies were mentioned as the most straightforward and easy-to-understand construct, which may be the reason why most of the studies undertaken to understand structural stigma investigated discrimination within policies [5–7]. However, the structures within a complex system such as a healthcare system consist of numerous interrelated components, and so ignoring the complexity and multilevel/multi-component structures restricts the understanding and methodological possibilities of evaluating the phenomenon. Hence, the current framework, although limited to healthcare systems, considers the complex structures across multiple interrelated domains. Through this framework, we can define structural stigma in healthcare systems as: *Inequities within health system structures (such as governance*, *infrastructure*, *resources*, *and service delivery) due to intended or unintended negative policies and practices that result in negative experiences of stigmatized populations and limit their access to quality healthcare*. This conceptualization may also contest the common assumption that ascertaining changes in structural stigma takes an extensive period and cannot be measured short term [1].

The **second** advantage of the framework is the representation of health systems structures within the domains, which makes it easier for health systems researchers and stakeholders to understand and measure the multifaceted phenomena. The most widely used framework in monitoring and evaluating health systems is provided by the World Health Organization (WHO) which has proposed health systems structures consisting of six building blocks to assess the health system structures and its performances: i) leadership/governance; ii) health financing; iii) health workforce; iv) information systems; v) medical infrastructures; and vi) health service delivery [30]. This building block framework has been used ubiquitously in health systems research mainly due to its simplicity and ability to provide a universal understanding of health systems structures and their functioning [31]. However, the framework has received several criticisms, most of which are focused on its limitation to capture the dynamic nature of the interlinked structures, and its lack of component that addresses the “demand” or outcome aspects of the health systems [31, 32]. Similar criticism was also made on the Livingston framework of measuring structural stigma by the experts during consultation workshops. They remarked that the six domains proposed in the framework were represented as existing in silos, which made it difficult for the experts to sort the indicators in different domains as they could be seen as causes and consequences of each other.

The current framework addresses some of these criticisms by demonstrating the interlinkages and dynamic nature of the domains. For example, the domains- discriminatory legal framework and policy environment, stigmatizing system infrastructure and resources, and stigmatizing attitudes and practices of health systems professionals (representing the governance, resources, infrastructures, financing, and health workforce of the WHO building blocks) are all interlinked as causes and consequences. These three domains collectively lead to inequitable and poor quality of care domain (representing the service delivery component of the WHO building blocks). This leads to the negative experiences of PWLEs, which represent the outcome domain within the framework. This rethinking of structural stigma in the healthcare system also addresses the critiques in recent literature on the tendency to overlook provider-induced stigma as an integral construct within the structural stigma phenomena [13]. Although some of the experts during our study suggested the removal of domain 3 (stigmatizing attitudes and practices of health system professionals) from the framework due to its similarity with interpersonal stigma, the majority (64% of total experts, and 80% of the PWLEs) shared that the domain was an integral part of system structure and hence needed to be included.

The **third** advantage is its universality in measuring health systems related to structural stigma and discrimination in multiple settings globally, including low resource settings in the Global South, while also providing flexibility to adapt to the context-specific needs and availability of data sources. The framework, as described above, conceptualizes structural stigma as inequities within the health system structures (governance and policies, resources, infrastructures, service delivery, etc.), which are universal in most health systems and hence can be implemented globally. However, the indicators identified within each domain may be subjected to variances depending on the health system context and availability of data structures. The indicators within domains, although identified through multiple rounds of consensus-building exercises with experts from different backgrounds (academics, policymakers, PWLEs) and wide geographic locations, may not apply to all health system settings. For example, the indicator “unavailability of mental health-related medications at health facilities”, may not apply to health systems in high-income settings but may apply only to health systems within LMICs, as some of our experts commented. This indicator can therefore be replaced by other indicators within the stigmatizing infrastructure and resources domain for health systems within high-income settings (e.g. higher cost of mental health medications/limited insurance coverage).

There are limitations to the framework and the study that needs to be considered. The first is the representativeness of expert panellists who completed the survey. Although we attempted to include panellists from wide geographic locations, the study had little to no representation from certain countries or cultural contexts such as the Latin America and Middle East regions. Similarly, despite efforts to reach PWLEs during the survey through the GMHPN which has networks in 45 countries, our study had fewer PWLE respondents during Round 2 (n = 5) and Round 3 (n = 15). This could be due to a lack of direct access to the GMHPN members, as the anonymous link to the survey could only be shared by the global office team. This limited our communication in terms of reminders and addressing questions/concerns. Another issue was the length and technical complexity of the Round 2 survey, where panellists had to select and rank indicators 3 times in each domain (for 3 criteria- appropriateness, usefulness, feasibility), and so the task had to be repeated a total of 15 times during the survey. Due to the low response rate in Round 2, we simplified the survey in Round 3 and asked the panellists to re-rank indicators (if they disagreed with the Round 2 results) only using the criteria of importance. This resulted in a higher response rate in Round 3, especially from PWLEs. Due to the anonymity of the survey, it was not feasible to explore attrition rates and to know how many participants completed all three rounds or participated only in some of the rounds of the Delphi process. This may have impacted the interpretation of the results from different rounds of the study.

The second study-related limitation is tied to the Delphi and other consensus building studies as a whole, where the subsequent rounds result in convergence of opinions as they mostly seek consensus on findings from previous rounds [33]. The strong consensus achieved on the third round of Delphi (re-ranking) exercise could be due to participants confirming the widely accepted ranking from second round. We tried to minimise this limitation by highlighting the anonymity of the survey, making the survey more user friendly in subsequent rounds so that it was easy for participants to navigate the survey, and by adding open feedback sections to get their views even if they chose not to change the ranking.

Third, although structural forms of stigma and discrimination are seen in wider social and political systems- such as the criminal justice, education, and employment systems [22], the current framework only addresses the structures within formal healthcare systems. The interlinkages and complexities of structures within healthcare systems also apply to between systems interlinkages and complexities, and so understanding the true nature of structural stigma against PWLEs may be incomplete with a focus on just healthcare systems. This concern was also raised by some of our panellists, who commented on the risk of the current framework pushing the medical model of mental healthcare and not highlighting the disability and social models. However, the scope of the current study was to delve deeper into the complexities and nuances of one system structure rather than providing a breadth of information from multiple system structures.

Fourth, the framework proposes domains and indicators based on the importance of understanding structural stigma and discrimination in the healthcare system. However, its applicability and utility remain unclear in the absence of methodological considerations and data sources. Hence, piloting of the framework in a healthcare system setting or multiple settings needs to be carried out to assess the applicability and utility and refine the framework.

## Conclusion

Experts in stigma research and PWLE, including respondents from LMICs, developed a measurement framework for mental health-related structural stigma in healthcare system settings by identifying indicators that matter most to stigma stakeholders (PWLEs, researchers, and policymakers). Through the process of a modified Delphi study consisting of 3 rounds, a measurement framework was developed that included 5 domains (1. discriminatory legal framework and policy environment; 2. stigmatizing system infrastructure and resource allocation; 3. aggregate stigma attitude and practices of individuals within healthcare systems; 4. inequitable/poor quality of care; and 5. negative experiences of PWLEs). Each domain is interlinked with the others and consists of the most salient indicators as ranked by the expert panellists. This framework helps to understand and monitor the complex nature of structural stigma against PWLEs in healthcare systems. The framework also aids in conceptualizing the definition of structural stigma in healthcare and framing it in terms of inequities within healthcare system structures that result in negative experiences of PWLEs and limit their access to quality healthcare. Such a conceptualization makes it easier to measure structural stigma in diverse healthcare system settings globally, while still providing flexibility to adapt to local structures and monitor short-term changes.

## Supporting information

S1 Table List of literatures and indicators identified in round 1. (XLSX)

S2 Table Full results table from Delphi Round 2 and Round 3 disaggregated by expert types. (DOCX)

S1 File Inclusivity in global research. (DOCX)
