# Supplementary material for: Development of a mental health-related structural stigma measurement framework in the healthcare system setting: A modified Delphi study
Source: PLoS One. 2025 Jan 31;20(1):e0316999. doi: 10.1371/journal.pone.0316999 (PMC11785284; doi:10.1371/journal.pone.0316999)
Supplement: S2 Table — (DOCX) [file pone.0316999.s002.docx]

**Table 1. Results of all indicators as ranked by all experts in Round 2**

| **Indicators in each domain** | **Appropriate** | | | **Useful** | | | **Feasible** | | | **Average Smith’s S*** |
| --- | --- | --- | --- | --- | --- | --- | --- | --- | --- | --- |
|  | Smith’s S | Frequency (%) | Average Rank | Smith’s S | Frequency (%) | Average Rank | Smith’s S | Frequency (%) | Average Rank |  |
| **Domain 1: Discriminatory legal framework and policy environment** |  |  |  |  |  |  |  |  |  |  |
| Unavailability of mental health policy and action plans | 0.49 | 61.5 | 1.75 | 0.49 | 63.5 | 1.93 | 0.63 | 76.9 | 1.72 | **0.54** |
| Exclusion of mental health from Universal Health Coverage | 0.36 | 53.8 | 2.43 | 0.25 | 38.5 | 2.35 | 0.38 | 55.8 | 2.27 | **0.33** |
| Exclusion of mental health from other national health policies and programs | 0.31 | 48.1 | 2.44 | 0.35 | 57.7 | 2.63 | 0.29 | 50 | 2.65 | **0.32** |
| Lack of involvement of People with Lived Experiences (PWLE) involvement in policy/program development | 0.37 | 55.8 | 2.43 | 0.29 | 50 | 2.65 | 0.25 | 44.2 | 2.82 | **0.30** |
| Discriminatory language or provision in mental health policy | 0.21 | 32.7 | 2.41 | 0.29 | 46.2 | 2.37 | 0.26 | 44.2 | 2.39 | **0.25** |
| Lack of coverage in national /community health insurance policies | 0.27 | 50 | 3.03 | 0.28 | 53.8 | 2.85 | 0.21 | 40.4 | 2.95 | **0.25** |
| Exclusion of mental health from disability or other socialwelfare policies | 0.25 | 57.7 | 3.33 | 0.20 | 42.3 | 3.18 | 0.23 | 44.2 | 3.08 | **0.23** |
| Differential rules or guidelines for PWLEs to access health services | 0.18 | 32.7 | 2.82 | 0.15 | 19.2 | 1.9 | 0.1 | 17.3 | 2.77 | **0.14** |
| Differential rules or guidelines for storage/access of information of PWLEs | 0.02 | 5.8 | 3.33 | 0.06 | 9.6 | 2.2 | 0.03 | 7.7 | 3.5 | **0.04** |
| Underfunded mental health policies | 0.006 | 1.9 | 3 |  |  |  |  |  |  | **0.002** |
| **Domain 2: Stigmatizing system infrastructure and resource allocation** |  |  |  |  |  |  |  |  |  |  |
| Insufficient funding for mh services & programs | 0.72 | 82 | 1.48 | 0.59 | 76 | 1.92 | 0.46 | 62 | 2.03 | **0.59** |
| Unavailability of trained mh human resources | 0.35 | 54 | 2.33 | 0.36 | 52 | 2.19 | 0.32 | 50 | 2.44 | **0.35** |
| MH indicators not included in national health information system | 0.21 | 38 | 2.94 | 0.21 | 40 | 2.9 | 0.32 | 46 | 2.17 | **0.24** |
| Differential quality of space/infrastructure for mh compared to other health services | 0.27 | 40 | 2.4 | 0.13 | 20 | 2.6 | 0.19 | 30 | 2.53 | **0.19** |
| Unaffordable services compared to other chronic conditions | 0.20 | 32 | 2.62 | 0.18 | 28 | 2.57 | 0.21 | 28 | 2.21 | **0.19** |
| Unavailability of mh medications at the health facilities | 0.13 | 28 | 3.57 | 0.19 | 34 | 3 | 0.16 | 34 | 3.12 | **0.16** |
| Insufficient funding for mh research | 0.19 | 34 | 2.82 | 0.14 | 24 | 2.75 | 0.09 | 16 | 2.50 | **0.14** |
| Number of mental health training conducted per year for general health workers | 0.06 | 14 | 3.71 | 0.15 | 26 | 2.69 | 0.21 | 30 | 2.13 | **0.14** |
| Systematically less hours allocated for mh training compared to other health training | 0.05 | 12 | 3.83 | 0.15 | 26 | 2.84 | 0.165 | 26 | 2.69 | **0.12** |
| Spaces that are undignified or non-conducive to recovery | 0.166 | 28 | 2.57 | 0.15 | 26 | 2.69 | 0.03 | 8 | 3.75 | **0.11** |
| Stigmatizing messages against mental health in information/education materials that are available | 0.08 | 20 | 3.7 | 0.06 | 18 | 3.78 | 0.07 | 20 | 3.4 | **0.07** |
| Mental health recording/reporting unsystematic and ad hoc compared to other health services | 0.05 | 16 | 4 | 0.08 | 14 | 2.5 | 0.06 | 14 | 3.26 | **0.06** |
| Unavailability of Information Education materials and guides for mental health | 0.028 | 6 | 3.66 | 0.03 | 8 | 3.25 | 0.09 | 20 | 3.1 | **0.05** |
| Unsystematic/poor supply chain management of MH medications compared to other medicines | 0.037 | 8 | 3.5 | 0.048 | 8 | 2.5 | 0.01 | 2 | 3 | **0.03** |
| **Domain 3: Aggregate stigma attitude and practices of individuals within healthcare systems** |  |  |  |  |  |  |  |  |  |  |
| Negative aggregate attitude/behavior of health and other staffs towards PWLEs | 0.58 | 76 | 1.94 | 0.59 | 78 | 1.81 | 0.49 | 72 | 2.13 | **0.55** |
| HWs not aware or knowledgeable about human rights of PWLEs | 0.42 | 74 | 2.65 | 0.45 | 72 | 2.33 | 0.53 | 82 | 2.19 | **0.47** |
| Less competency in dealing with mh patients | 0.36 | 62 | 2.61 | 0.47 | 70 | 2.17 | 0.42 | 60 | 2 | **0.42** |
| Culture of not involving PWLEs in decision making | 0.49 | 68 | 2.03 | 0.34 | 66 | 2.76 | 0.33 | 52 | 2.27 | **0.39** |
| Culture of stigmatizing MH staffs by other health/non-health professionals in health systems | 0.29 | 58 | 2.96 | 0.27 | 44 | 2.54 | 0.24 | 40 | 2.3 | **0.27** |
| Withholding information from PWLEs | 0.12 | 20 | 2.4 | 0.05 | 10 | 3.2 | 0.09 | 18 | 2.88 | **0.09** |
| Culture of taboo/non-disclosure of(own) mental illness(history)among healthcare staff | 0.01 | 2 | 2 | 0.02 | 2 | 1 | 0.02 | 2 | 1 | **0.01** |
| Culture of "Othering" PWLE (as different, defective,mad_etc.) | 0.02 | 2 | 1 | 0.01 | 2 | 2 | 0 | 0 | 0 | **0.01** |
| **Domain 4: Inequitable and poor quality of care** |  |  |  |  |  |  |  |  |  |  |
| Involuntary/compulsory treatment of PWLEs | 0.35 | 44 | 1.90 | 0.27 | 43 | 2.52 | 0.32 | 42 | 2 | **0.31** |
| Unavailability of evidence based MH services | 0.27 | 42 | 2.52 | 0.29 | 41 | 2.2 | 0.31 | 49 | 2.58 | **0.29** |
| Separation of mh services from Primary health or basic health services | 0.26 | 37 | 2.22 | 0.26 | 43 | 2.52 | 0.34 | 49 | 2.20 | **0.28** |
| Lack of multi-sectoral collaboration within health systems for mental health compared to other health conditions | 0.34 | 53 | 2.5 | 0.32 | 51 | 2.52 | 0.20 | 32 | 2.56 | **0.28** |
| Lack of clear referral pathway system | 0.19 | 37 | 3.05 | 0.18 | 37 | 3.16 | 0.24 | 42 | 2.62 | **0.21** |
| Lack of sufficient Out Patient Care for people with severe MH conditions | 0.14 | 29 | 3.28 | 0.16 | 29 | 2.92 | 0.22 | 36 | 2.72 | **0.17** |
| PWLEs Not able to easily access disability services and grants | 0.14 | 27 | 3.15 | 0.21 | 31 | 2.26 | 0.08 | 20 | 3.2 | **0.14** |
| Paternalistic/non-collaborative approaches | 0.21 | 35 | 2.70 | 0.13 | 22 | 2.90 | 0.08 | 12 | 2.5 | **0.14** |
| Lack of access to rehabilitation services for PWLEs | 0.12 | 20 | 2.7 | 0.19 | 29 | 2.42 | 0.09 | 18 | 2.88 | **0.14** |
| Segregated health and social care systems compared to other health conditions | 0.15 | 22 | 2.36 | 0.09 | 18 | 2.88 | 0.11 | 18 | 2.55 | **0.12** |
| Interaction with justice/security during treatment | 0.08 | 16 | 3.12 | 0.09 | 12 | 2.16 | 0.11 | 16 | 2.12 | **0.09** |
| Exclusion of PWLEs from screening services | 0.08 | 14 | 2.71 | 0.09 | 12 | 1.83 | 0.10 | 16 | 2.5 | **0.09** |
| Delay in onset of mental health condition to start of treatment | 0.10 | 18 | 2.66 | 0.08 | 18 | 3.33 | 0.09 | 16 | 2.7 | **0.09** |
| Diagnostic & Treatment overshadowing | 0.07 | 16 | 3.37 | 0.07 | 10 | 1.8 | 0.08 | 20 | 3.2 | **0.07** |
| Medication errors for mental health care compared to general health care | 0.01 | 2 | 3 | 0 | 0 | 0 | 0 | 0 | 0 | **0.004** |
| **Domain 5: negative experiences of PWLEs** |  |  |  |  |  |  |  |  |  |  |
| PWLE low satisfaction of care received for mh | 0.33 | 45 | 2.18 | 0.35 | 47 | 1.82 | 0.51 | 65 | 1.81 | **0.39** |
| PWLE negative interaction with HW/administrators | 0.49 | 65 | 2.03 | 0.31 | 49 | 2.45 | 0.29 | 49 | 2.58 | **0.36** |
| PWLE higher Out of pocket expenses for mh services compared to other health services | 0.24 | 45 | 3.09 | 0.37 | 53 | 2.11 | 0.34 | 44 | 1.77 | **0.32** |
| PWLE lack of ease of access of mh services vs physical services | 0.28 | 45 | 2.41 | 0.33 | 49 | 2.08 | 0.27 | 46 | 2.47 | **0.24** |
| PWLE insufficiently informed about their condition or treatment | 0.31 | 49 | 2.54 | 0.19 | 35 | 2.76 | 0.18 | 26 | 2.23 | **0.23** |
| PWLE experience of undignified treatment process | 0.23 | 39 | 2.52 | 0.21 | 37 | 2.5 | 0.15 | 30 | 2.86 | **0.20** |
| PWLE lack of ease of access to social services when needed | 0.19 | 33 | 2.68 | 0.17 | 25 | 2.25 | 0.15 | 24 | 2.66 | **0.17** |
| PWLE feeling devalued and infantilized by HW/admins | 0.15 | 31 | 3.06 | 0.15 | 29 | 2.85 | 0.15 | 26 | 2.53 | **0.15** |
| PWLE experience of hasty referrals or no referrals (even when needed) | 0.06 | 18 | 3.44 | 0.09 | 22 | 3.18 | 0.13 | 26 | 2.77 | **0.10** |
| PWLE experience of being hastily diagnosed and treated | 0.15 | 22 | 2.45 | 0.07 | 8 | 1.5 | 0.05 | 10 | 3 | **0.09** |

**Table 2. Results of Round 2 all indicators as ranked PWLE experts only (n=5)**

|  |  |  |  |  |
| --- | --- | --- | --- | --- |
| **Indicators in each domain** | **Smith’s S Appropriate** | **Smith’s S Useful** | **Smith’s S Feasible** | **Average Smith’s S*** |
| **Domain 1: Discriminatory legal framework and policy environment** |  |  |  |  |
| Unavailability of mental health policy and action plans | 0.48 | 0.61 | 0.68 | 0.59 |
| Exclusion of mental health from Universal Health Coverage | 0.32 | 0.17 | 0.22 | 0.24 |
| Exclusion of mental health from other national health policies and programs | 0.25 | 0.26 | 0.28 | 0.26 |
| Lack of involvement of People with Lived Experiences (PWLE) involvement in policy/program development | 0.6 | 0.6 | 0.57 | 0.59 |
| Discriminatory language or provision in mental health policy | 0.17 | 0.24 | 0.49 | 0.32 |
| Lack of coverage in national /community health insurance policies | 0.12 | 0.22 | 0.16 | 0.17 |
| Exclusion of mental health from disability or other socialwelfare policies | 0.30 | 0.2 | 0.08 | 0.19 |
| Differential rules or guidelines for PWLEs to access health services | 0 | 0 | 0 | 0 |
| Differential rules or guidelines for storage/access of information of PWLEs | 0.06 | 0 | 0 | 0.02 |
| Underfunded mental health policies | 0 | 0 | 0 | 0 |
| **Domain 2: Stigmatizing system infrastructure and resource allocation** |  |  |  |  |
| Insufficient funding for mh services & programs | 0.46 | 0.81 | 0.76 | 0.68 |
| Unavailability of trained mh human resources | 0.22 | 0.49 | 0.24 | 0.32 |
| MH indicators not included in national health information system | 0.36 | 0.31 | 0.4 | 0.35 |
| Differential quality of space/infrastructure for mh compared to other health services | 0.08 | 0.04 | 0.04 | 0.05 |
| Unaffordable services compared to other chronic conditions | 0.42 | 0.2 | 0.41 | 0.34 |
| Unavailability of mh medications at the health facilities | 0.08 | 0.12 | 0.16 | 0.12 |
| Insufficient funding for mh research | 0.36 | 0.08 | 0.08 | 0.173 |
| Number of mental health training conducted per year for general health workers | 0.09 | 0.30 | 0.24 | 0.21 |
| Systematically less hours allocated for mh training compared to other health training | 0 | 0.08 | 0 | 0.027 |
| Spaces that are undignified or non-conducive to recovery | 0.41 | 0.16 | 0.04 | 0.20 |
| Stigmatizing messages against mental health in information/education materials that are available | 0 | 0 | 0.06 | 0.02 |
| Mental health recording/reporting unsystematic and ad hoc compared to other health services | 0 | 0 | 0.16 | 0.05 |
| Unavailability of Information Education materials and guides for mental health | 0 | 0 | 0 | 0 |
| Unsystematic/poor supply chain management of MH medications compared to other medicines | 0 | 0 | 0 | 0 |
| **Domain 3: Aggregate stigma attitude and practices of individuals within healthcare systems** |  |  |  |  |
| Negative aggregate attitude/behavior of health and other staffs towards PWLEs | 0.72 | 0.66 | 0.6 | 0.66 |
| HWs not aware or knowledgeable about human rights of PWLEs | 0.51 | 0.68 | 0.61 | 0.60 |
| Less competency in dealing with mh patients | 0.14 | 0.19 | 0.11 | 0.15 |
| Culture of not involving PWLEs in decision making | 0.61 | 0.48 | 0.55 | 0.55 |
| Culture of stigmatizing MH staffs by other health/non-health professionals in health systems | 0.38 | 0.33 | 0.31 | 0.34 |
| Withholding information from PWLEs | 0.12 | 0.04 | 0 | 0.05 |
| Culture of taboo/non-disclosure of(own) mental illness(history)among healthcare staff | 0 | 0 | 0 | 0 |
| Culture of "Othering" PWLE (as different, defective,mad_etc.) | 0 | 0 | 0 | 0 |
| **Domain 4: Inequitable and poor quality of care** |  |  |  |  |
| Involuntary/compulsory treatment of PWLEs | 0.8 | 0.48 | 0.66 | 0.64 |
| Unavailability of evidence-based MH services | 0.12 | 0.24 | 0.29 | 0.20 |
| Separation of mh services from Primary health or basic health services | 0.05 | 0.13 | 0.22 | 0.13 |
| Lack of multi-sectoral collaboration within health systems for mental health compared to other health conditions | 0.16 | 0.12 | 0.36 | 0.21 |
| Lack of clear referral pathway system | 0.2 | 0.04 | 0.04 | 0.93 |
| Lack of sufficient Out Patient Care for people with severe MH conditions | 0.28 | 0.28 | 0.35 | 0.30 |
| PWLEs Not able to easily access disability services and grants | 0.04 | 0.16 | 0.08 | 0.09 |
| Paternalistic/non-collaborative approaches | 0.39 | 0.31 | 0.2 | 0.30 |
| Lack of access to rehabilitation services for PWLEs | 0.22 | 0.3 | 0.15 | 0.22 |
| Segregated health and social care systems compared to other health conditions | 0.08 | 0 | 0 | 0.02 |
| Interaction with justice/security during treatment | 0.16 | 0.12 | 0.36 | 0.21 |
| Exclusion of PWLEs from screening services | 0.06 | 0.133 | 0.133 | 0.11 |
| Delay in onset of mental health condition to start of treatment | 0.13 | 0.10 | 0.06 | 0.10 |
| Diagnostic & Treatment overshadowing | 0 | 0 | 0 | 0 |
| Medication errors for mental health care compared to general health care | 0 | 0 | 0 | 0 |
| **Domain 5: negative experiences of PWLEs** |  |  |  |  |
| PWLE low satisfaction of care received for mh | 0.48 | 0.56 | 0.66 | 0.57 |
| PWLE negative interaction with HW/administrators | 0.66 | 0.08 | 0.30 | 0.35 |
| PWLE higher Out of pocket expenses for mh services compared to other health services | 0.37 | 0.6 | 0.45 | 0.47 |
| PWLE lack of ease of access of mh services vs physical services | 0.11 | 0.21 | 0.21 | 0.18 |
| PWLE insufficiently informed about their condition or treatment | 0.12 | 0.16 | 0.2 | 0.16 |
| PWLE experience of undignified treatment process | 0.39 | 0.47 | 0.24 | 0.36 |
| PWLE lack of ease of access to social services when needed | 0.1 | 0.1 | 0.18 | 0.12 |
| PWLE feeling devalued and infantilized by HW/admins | 0.37 | 0.33 | 0.16 | 0.28 |
| PWLE experience of hasty referrals or no referrals (even when needed) | 0 | 0.08 | 0.08 | 0.05 |
| PWLE experience of being hastily diagnosed and treated | 0 | 0 | 0 | 0 |

**Table 3: Results of all indicators as ranked by all experts and PWLE experts only for Round 3**

|  | **All experts (n=58)** | | | **PWLE experts only (n=15)** | | |
| --- | --- | --- | --- | --- | --- | --- |
| **Indicators in each domain** | **Smith’s S** | **Average rank** | **Kendall’s W (p-value)** | **Smith’s S** | **Average rank** | **Kendall’s W (p-value)** |
| **Domain 1** |  |  |  |  |  |  |
| Unavailability of mental health policy and action plans | 0.96 | 1.32 | 0.87 (<0.001) | 0.93 | 1.66 | 0.82 (<0.001) |
| Exclusion of mental health from Universal Health Coverage | 0.86 | 2.33 |  | 0.81 | 2.86 |  |
| Exclusion of mental health from other national health policies and programs | 0.75 | 3.44 |  | 0.75 | 3.46 |  |
| Lack of involvement of People with Lived Experiences (PWLE) in policy/program development | 0.71 | 3.91 |  | 0.74 | 3.60 |  |
| Discriminatory language or provision in mental health policy | 0.61 | 4.84 |  | 0.64 | 4.53 |  |
| Lack of coverage in national /community health insurance policies | 0.52 | 5.79 |  | 0.50 | 5.93 |  |
| Exclusion of mental health from disability or other social welfare policies | 0.43 | 6.70 |  | 0.46 | 6.33 |  |
| Differential rules or guidelines for PWLEs to access health services | 0.31 | 7.9 |  | 0.34 | 7.60 |  |
| Differential rules or guidelines for storage/access of information of PWLEs | 0.21 | 8.82 |  | 0.2 | 9 |  |
| Underfunded mental health policies | 0.11 | 9.89 |  | 0.1 | 10 |  |
|  |  |  |  |  |  |  |
| **Domain 2** |  |  |  |  |  |  |
| Insufficient funding for mh services & programs | 0.97 | 1.32 | 0.88 (<0.001) | 0.91 | 2.2 | 0.79 (<0.001) |
| Unavailability of trained mh human resources | 0.91 | 2.22 |  | 0.87 | 2.7 |  |
| MH indicators not included in national health information system | 0.82 | 3.43 |  | 0.81 | 3.6 |  |
| Unaffordable services compare to other chronic conditions  Insufficient funding for mh research | 0.75 | 4.5 |  | 0.78 | 4.0 |  |
| Differential quality of space/infrastructure for mh compared to other health services | 0.69 | 5.24 |  | 0.70 | 5.0 |  |
| Unavailability of mh medications at the health facilities | 0.65 | 5.87 |  | 0.66 | 5.7 |  |
| Number of mental health training conducted per year for general health workers | 0.57 | 6.93 |  | 0.56 | 7.1 |  |
| Systematically less hours allocated for mh training compared to other health training | 0.43 | 8.96 |  | 0.53 | 7.5 |  |
| Spaces that are undignified or non-conducive to recovery | 0.37 | 9.74 |  | 0.42 | 9.0 |  |
| Stigmatizing messages against mental health in information/education materials that are available | 0.29 | 10.9 |  | 0.38 | 9.6 |  |
| Mental health recording/reporting unsystematic and ad hoc compared to other health services | 0.22 | 11.87 |  | 0.29 | 10.9 |  |
|  |  |  |  | 0.21 | 12.0 |  |
| Unsystematic/poor supply chain management of MH medications compared to other medicines | 0.10 | 13.51 |  | 0.17 | 12.6 |  |
| Unavailability of Information Education materials and guides for mental health | 0.15 | 12.81 |  | 0.15 | 12.8 |  |
|  |  |  |  |  |  |  |
| **Domain 3** |  |  |  |  |  |  |
| Negative aggregate attitude/behavior of health and other staffs towards PWLEs | 0.96 | 1.29 | 0.86 (<0.001) | 0.97 | 1.2 | 0.84 (<0.001) |
| HWs not aware or knowledgeable about human rights of PWLEs | 0.86 | 2.20 |  | 0.83 | 2.4 |  |
| Less competency in dealing with mh patients | 0.77 | 3 |  | 0.78 | 2.9 |  |
| Culture of not involving PWLEs in decision making | 0.65 | 4.13 |  | 0.63 | 4.2 |  |
| Culture of stigmatizing MH staffs by other health/non-health professionals in health systems | 0.53 | 5.19 |  | 0.50 | 5.4 |  |
| Withholding information from PWLEs | 0.45 | 5.91 |  | 0.48 | 5.6 |  |
| Culture of taboo/non-disclosure of(own) mental illness(history)among healthcare staff | 0.25 | 7.72 |  | 0.36 | 6.7 |  |
| Culture of "Othering" PWLE (as different, defective,mad_etc.) | 0.14 | 8.74 |  | 0.28 | 7.4 |  |
| **Domain 4** |  |  |  |  |  |  |
| Involuntary/compulsory treatment of PWLEs | 0.96 | 1.56 | 0.83 (<0.001) | 0.96 | 1.5 | 0.84 (<0.001) |
| Unavailability of evidence based MH services | 0.87 | 2.82 |  | 0.88 | 2.7 |  |
| Separation of mh services from Primary health or basic health services | 0.79 | 4.08 |  | 0.79 | 4.1 |  |
| Lack of clear referral pathway system | 0.74 | 4.91 |  | 0.74 | 4.8 |  |
| Lack of multi-sectoral collaboration within health systems for mental health compared to other health conditions | 0.72 | 5.13 |  | 0.71 | 5.3 |  |
| Lack of sufficient Out Patient Care for people with severe MH conditions | 0.68 | 5.74 |  | 0.68 | 5.8 |  |
| PWLEs Not able to easily access disability services and grants | 0.63 | 6.48 |  | 0.61 | 6.8 |  |
| Interaction with justice/security during treatment | 0.63 | 10.56 |  | 0.59 | 7.1 |  |
| Paternalistic/non-collaborative approaches | 0.58 | 7.24 |  | 0.47 | 8.9 |  |
| Lack of access to rehabilitation services for PWLEs | 0.48 | 8.74 |  | 0.42 | 9.6 |  |
| Segregated health and social care systems compared to other health conditions | 0.41 | 9.82 |  | 0.28 | 9.6 |  |
| Exclusion of PWLEs from screening services | 0.26 | 12 |  | 0.20 | 11.8 |  |
| Delay in onset of mental health condition to start of treatment | 0.22 | 12.65 |  | 0.14 | 12.9 |  |
| Diagnostic & Treatment overshadowing | 0.13 | 13.9 |  | 0.14 | 13.8 |  |
| Medication errors for mental health care compared to general health care | 0.11 | 14.25 |  | 0.07 | 14.9 |  |
| **Domain 5** |  |  |  |  |  |  |
| PWLE low satisfaction of care received for mh | 0.96 | 1.37 | 0.87 (<0.001) | 0.96 | 1.3 | 0.84 (<0.001) |
| PWLE negative interaction with HW/administrators | 0.87 | 2.25 |  | 0.88 | 2.2 |  |
| PWLE higher Out of pocket expenses for mh services compared to other health services | 0.76 | 3.31 |  | 0.76 | 3.4 |  |
| PWLE lack of ease of access of mh services vs physical services | 0.69 | 4.01 |  | 0.61 | 4.8 |  |
| PWLE insufficiently informed about their condition or treatment | 0.58 | 5.12 |  | 0.59 | 5.0 |  |
| PWLE experience of undignified treatment process | 0.53 | 5.62 |  | 0.58 | 5.1 |  |
| PWLE lack of ease of access to social services when needed | 0.42 | 6.79 |  | 0.44 | 6.6 |  |
| PWLE feeling devalued and infantilized by HW/admins | 0.31 | 7.82 |  | 0.32 | 7.8 |  |
| PWLE experience of hasty referrals or no referrals (even when needed) | 0.21 | 8.84 |  | 0.22 | 8.7 |  |
| PWLE experience of being hastily diagnosed and treated | 0.11 | 9.82 |  | 0.11 | 9.8 |  |
